# Editorial: 3D models in cancer research: bridging tumor biology and personalized medicine

**DOI:** 10.3389/fcell.2026.1949643

**Published:** 2026-08-26

**Authors:** Yahima Frión-Herrera, Ariane Rocha Bartolomeu, Daniela Gabbia, Jeremy C. Simpson

**Affiliations:** 1 Department of Medicine, School of Medicine and Surgery, University of Padua, Padua, Italy; 2 Department of Cancer Prevention and Control, Fox Chase Cancer Center, Philadelphia, PA, United States; 3 Department of Pharmaceutical and Pharmacological Sciences, University of Padua, Padua, Italy; 4 Cell Screening Laboratory, UCD School of Biology & Environmental Science, University College Dublin, Dublin, Ireland

**Keywords:** 3D models, organoid, organ-on-a-chip, patient-derived models, spheroid, tumor microenvironment

Cancer remains one of the leading causes of morbidity and mortality worldwide, largely due to its remarkable biological complexity and inter- and intratumoral heterogeneity (Bray et al., 2024). These features have challenged the development of effective therapeutic strategies and highlighted the limitations of conventional experimental models. While two-dimensional (2D) cell cultures have substantially advanced our understanding of cancer biology, they fail to faithfully reproduce the intricate architecture of tumors, the dynamic interactions between malignant and stromal cells, and the biochemical and mechanical gradients that characterize the tumor microenvironment (TME). Although animal models remain indispensable in translational oncology, species-specific differences and ethical concerns reinforce the need for more physiologically relevant and advanced human-based experimental systems.

In this context, three-dimensional (3D) *in vitro* models, including multicellular spheroids, organoids, organ-on-chip systems, and advanced co-culture platforms, have all emerged as novel tools for studying tumor biology and accelerating precision oncology. By better preserving tissue architecture, cellular heterogeneity, extracellular matrix interactions, and microenvironmental cues, these models bridge the gap between conventional *in vitro* systems and human disease. Furthermore, they contribute to the implementation of the 3Rs principles (Replacement, Reduction, and Refinement), supporting the progressive reduction of animal experimentation while improving the predictive value of preclinical studies.

The articles collected in this Research Topic illustrate how the field has evolved to exploit these models for mechanistic studies, drug development, nanomedicine, and personalized therapeutic approaches. Rather than representing isolated advances, the contributions collectively depict a rapidly maturing research landscape in which increasingly sophisticated 3D models are becoming indispensable components of modern cancer research (Andersson-Rolf and Clevers, 2026).

One major emerging theme is the progressive refinement of 3D models as faithful representations of tumor biology (Cordeiro et al.). Recent developments in organoid technology have enabled the incorporation of immune and stromal components, providing more physiologically relevant models to investigate tissue homeostasis, inflammation, and disease (Andersson-Rolf and Clevers, 2026). Kumar et al. show that cervical cancer cells cultured in 3D acquire a transcriptomic profile characterized by enhanced immune signalling, angiogenesis, extracellular matrix remodelling, and HPV-associated pathways (Kumar et al.). Moving beyond transcriptomic characterization, Fernández-Domínguez et al. demonstrate that cancer stem cell-enriched 3D cultures release greater amounts of biologically active cell-free DNA, providing new insights into tumor heterogeneity and the potential contribution of stem-like populations to disease progression (Fernández-Domínguez et al.). Together, these studies emphasize that 3D organization is not merely a structural feature, but an active regulator of cancer biology, with tissue architecture profoundly influencing cancer cell behaviour.

The importance of reproducing the tumor microenvironment is further reinforced by several complementary reviews. Yang et al. describe how immune-competent tumor organoids preserve stromal and immune components, useful to investigate immune evasion and immunotherapeutic responses (Yang et al.). Focusing on the liver, Pastore et al. discuss the applications of spheroids and organoids in primary liver cancers (Pastore et al.), while Montagner et al. extend this concept to cholangiocarcinoma by comparing multiple 3D platforms, including spheroids, organoids, tumor-on-chip technologies and co-culture systems, as complementary rather than competing approaches (Montagner et al.).

Interestingly, although outside the strict oncology field, the review by Kong et al. on bone organoids illustrates the rapid technological evolution of organoid engineering and biomaterials; developments that are expected to accelerate the generation of increasingly complex cancer models incorporating vascular, stromal, and tissue-specific components (Kong et al.). Collectively, these contributions suggest that reproducing tumor complexity requires a toolbox of complementary 3D platforms tailored to different biological questions rather than a single ideal model.

A second major theme of the collection concerns the transition of 3D models from experimental systems toward clinically relevant platforms. Patient-derived organoids have become one of the most promising tools for precision oncology since they preserve the molecular heterogeneity of individual tumors allowing rapid *ex vivo* therapeutic testing. Liu et al. comprehensively review the current state of patient-derived lung cancer organoids, highlighting their applications in drug screening, biobanking, resistance studies, and their integration with emerging technologies such as single-cell sequencing, organoid-on-chip platforms, and 3D bioprinting (Liu et al.). Providing experimental evidence for this translational potential, Yun et al. demonstrate that lung adenocarcinoma organoids closely reproduce chemotherapy responses, resistance evolution, and molecular alterations observed *in vivo*, supporting their use for individualized treatment selection (Wuyang Yun et al.). These findings complement the observations of Kumar et al., who illustrate how 3D cultures better preserve clinically relevant molecular programs than conventional monolayers, collectively reinforcing the notion that patient-derived 3D systems may substantially improve the predictive value of preclinical drug evaluation (Kumar et al.).

The versatility of 3D models is further illustrated by their application to nanomedicine and advanced therapeutic delivery. Cybulski et al. demonstrate that nanoparticle accumulation and penetration within tumor spheroids are strongly influenced by particle size, morphology, and surface charge, highlighting how complex three-dimensional architectures reveal transport limitations that cannot be appreciated in 2D cultures (Cybulski et al.). Chalkley et al. expand this perspective by reviewing how high-resolution fluorescence microscopy and high-content imaging can be integrated with spheroids and organoids to quantitatively monitor nanoparticle trafficking and intracellular drug delivery (Chalkley et al.). Together, these studies highlight the growing convergence between bioengineering, advanced imaging, and 3D biology, illustrating how technological innovation is driving increasingly quantitative and predictive preclinical platforms.

An interesting aspect is how the concept of 3D extends beyond cell culture itself. While most contributions focus on biologically relevant *in vitro* models, Su et al. demonstrate the prognostic value of integrating three-dimensional quantitative PET/CT imaging with clinicopathological parameters in non-small cell lung cancer (Su et al.). Although based on a different application of 3D technologies, this study reflects the same overarching goal shared by all contributions: generating multidimensional, clinically meaningful representations of tumor biology capable of improving diagnosis, prognosis, and therapeutic decision-making.

This Research Topic shows a field undergoing a profound transformation. The discussion is no longer centered on whether 3D models are superior to conventional cultures, but rather on how these systems can be optimized, standardized, and integrated with complementary technologies to address increasingly sophisticated biological and clinical questions. Patient-derived organoids, multicellular co-culture systems, tumor-on-chip platforms, quantitative imaging, nanomedicine, artificial intelligence, spatial and single-cell omics, and advanced bioengineering are progressively converging toward the development of comprehensive experimental platforms capable of reproducing tumor complexity with unprecedented fidelity.

Despite these remarkable advances, important challenges remain. Standardization of culture protocols, incorporation of functional vascular and immune compartments, reduction of inter-laboratory variability, scalability for high-throughput applications, and the establishment of harmonized validation criteria will all be essential for broad clinical implementation. Future efforts will likely focus on generating immune-competent and vascularized organoids, integrating patient-derived models with real-time imaging and computational prediction, and developing regulatory frameworks that facilitate the adoption of these systems in drug development and precision oncology.

Overall, 3D cancer models have evolved from innovative experimental tools into central platforms for understanding tumor biology and guiding translational research. By bringing together expertise from cancer biology, bioengineering, imaging sciences, biomaterials, nanotechnology, and computational biology, the contributions collected here provide a compelling vision of the future of cancer research in order to accelerate therapeutic discovery and help deliver truly personalized medicine.
